# Perinephric abscess caused by ruptured retrocecal appendix: MDCT demonstration

**DOI:** 10.4103/0974-7796.62923

**Published:** 2010

**Authors:** Nisar Ahmad Wani, Mir Farooq, Tariq Gojwari, Tasleem Kosar

**Affiliations:** Department of Radiology, Sher-I-Kashmir Institute of Medical Sciences (SKIMS), Srinagar, J&K, India

**Keywords:** Perinephric abscess, retrocecal appendicitis, multidetector-row CT-MDCT

## Abstract

Acute appendicitis may occasionally become extraordinarily complicated and life threatening yet difficult to diagnose. One such presentation is described in a 60-year-old man who was brought to the hospital due to right lumbar pain and fever for the last 15 days. Ultrasonography showed a right perinephric gas and fluid collection. Abdominal computed tomography with multidetector-row CT (MDCT) revealed gas-containing abscess in the right retroperitoneal region involving the perinephric space, extending from the lower pole of the right kidney up to the bare area of the liver. Inflamed retrocecal appendix was seen on thick multiplanar reformat images with its tip at the lower extent of the abscess. Laparotomy and retroperitoneal exploration were performed immediately and a large volume of foul smelling pus was drained. A ruptured retrocecal appendix was confirmed as the cause of the abscess.

## INTRODUCTION

Acute appendicitis is a disease commonly encountered in daily practice, and with timely diagnosis and management a very low morbidity and mortality rate can be achieved. Generally, in acute appendicitis, high resolution, graded compression ultrasonography can help the diagnosis. However, USG is less sensitive in cases with perforated appendicitis, whereas contrast-enhanced computed tomography particularly multidetector CT (MDCT) with thick coronal multiplanar reformat (MPR) images has better sensitivity and specificity in this clinical setting. Diagnostic imaging with either USG or CT may improve patient care by lowering the false-negative appendectomy rate. Ruptured retrocecal appendicitis may rarely result in retroperitoneal abscess, which may not be associated with typical abdominal signs and symptoms. Multidetector CT with MPRs very well depicts the extent of the abscess and inflamed appendix as its cause. This complication should not be overlooked in order to avoid further sequelae. We present here a rare case of ruptured retrocecal appendicitis with extensive formation of retroperitoneal abscess in right perinephric space emphasizing the role of MDCT in the diagnosis of ruptured appendicitis with this complication.

## CASE REPORT

**Figure 1:**
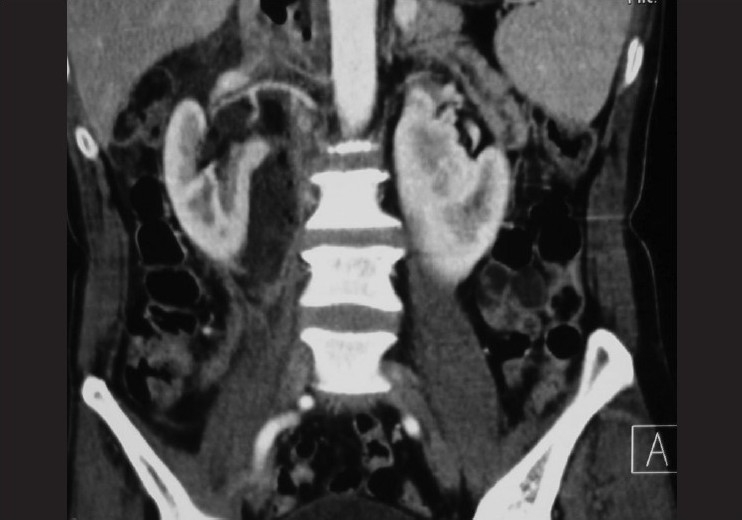
Coronal thick MPR CT image showing right perinephric abscess and inflammed appendix

**Figure 2:**
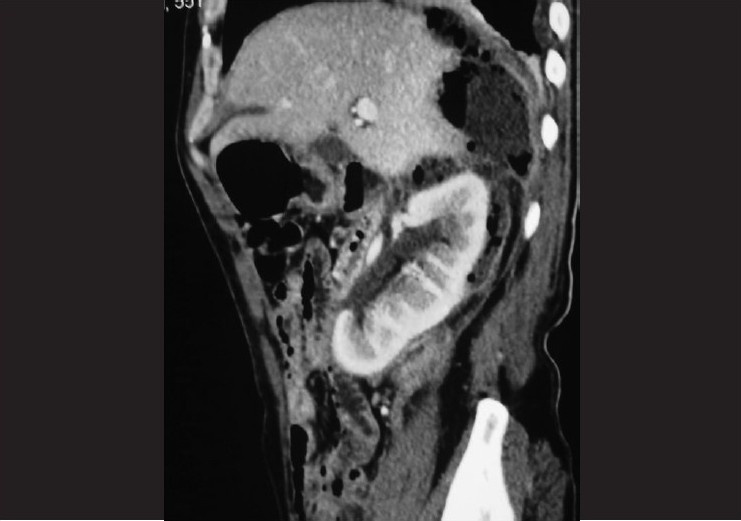
Sagittal reformat CT image showing right perinephric abscess reaching superiorly upto bare area of liver with inflammed appendix at its lower end below right kidney

**Figure 3:**
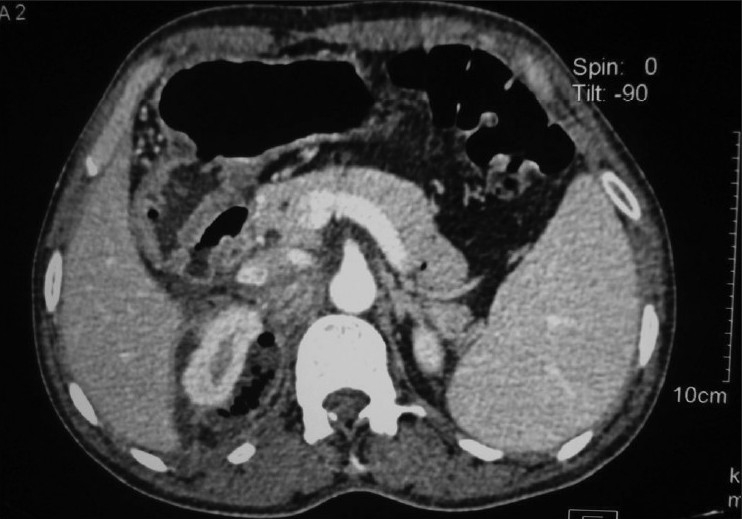
Axial CT image through the upper pole of right kidney showing perinephric abscess reaching posterior to IVC

A 60-year old non-diabetic, normotensive male patient presented to the emergency department with history of pain in the right lumbar region and fever for the last 15 days. There was no history of trauma, lower abdominal pain, dysuria or bowel disturbance. Physical examination on admission revealed an acute ill-looking man with a body temperature of 39°C. He was slightly anemic in appearance and breathed both shallowly and rapidly at a rate of 28 breathes/min. Palpation of the abdomen did not reveal any focal or diffuse tenderness; there was no organomegaly. Patient had tender right renal angle with some evidence of local erythema. Laboratory data indicated leukocytosis with predominant neutrophils. The hemoglobin level was slightly decreased to the level of 11 g/dL and his platelet count was normal. The C-reactive protein level and ESR were elevated. Urine examination showed 6–8 pus cells, however urine culture was sterile. Ultrasonography showed a mixed echotexture collection with gas shadows around the right kidney with evidence of horse-shoe kidney. There was no renal calculus, abscess or hydronephrosis. There was no intraperitoneal collection. Multidetector row CT of abdomen was performed to determine the nature and extent of the right perinephric collection [Figure 1‐3]. Axial images with sagittal and coronal plane reformations showed a fluid and air collection around the right kidney involving the perinephric space extending up to the bare area of the liver. There was right pleural effusion. There was no extension across the midline; psoas muscle was normal. On thick coronal MPR images, inflamed and collapsed appendix was seen with its base in the caecum and its tip at the lower extent of the right perinephric abscess. These findings were consistent with ruptured retrocecal appendicitis resulting in right perinephric abscess. Patient was operated and about 1 L of foul smelling pus was drained from the retroperitoneum around right kidney. Appendicectomy was performed and ruptured inflamed appendix was seen in the retroperitoneum around the lower extent of the abscess.

## DISCUSSION

Acute appendicitis is the most common abdominal emergency worldwide. Typically the patient presents with acute onset vague abdominal pain that gradually shifts to right iliac fossa with signs of peritoneal irritation or irritation of adjacent psoas or obturator muscle. Diagnosis is straightforward in a typical case, which demands prompt laparotomy. However, in equivocal cases, imaging with ultrasound and or computed tomography may decrease the rate of negative laparotomy.[1,2,3,4] Inflamed appendix may rupture and result in adjacent intraperitoneal abscesses in right iliac fossa or pelvis. However, formation of retroperitoneal abscesses involving thigh, psoas muscle, perinephric space or even the lateral abdominal wall, though rare but reported in the literature, remains one of the most serious complications of acute appendicitis and is always associated with perforation of a retrocecal appendix with delayed diagnosis and treatment.[1,2,4,5,6,7] Most of the patients that have been reported in the literature are adults, about one-third of them being older than 65 years. These patients do not present with the classical symptoms of acute appendicitis at the onset of the disease. The average interval between the onset of symptoms and diagnosis that has been reported is more than 15 days.[8] The most effective diagnostic tool in this subgroup of patients is computed tomography (CT).[6] Isolated retroperitoneal abscess formation has always been described in cases with perforated retrocecal appendicitis, which in fact is the commonest location of normal appendix.[8]

Retroperitoneal compartments described include perinephric space, anterior and posterior pararenal spaces and psoas compartment.[9] Perinephric space communicates with the pelvic retroperitoneal space inferiorly and continues superiorly reaching the bare area of the liver on the right side.[9] In our case, the extent of right perinephric space was very well depicted by the abscess collection, which besides surrounding the right kidney extended from the tip of appendix inferiorly up to the bare area of the liver superiorly. True extent of the retroperitoneal abscess is well defined by the MDCT, which besides providing the extent of the abscess in different orthogonal planes can demonstrate a reconstructed coronal image that is of great help for surgical planning because it simulates the operation field. In addition, the drainage of abscess can be achieved by percutaneous and retroperitoneal approach or by laparotomy based on CT findings.[2,8] Coronal thick MPR images have been reported to depict the inflamed appendix most beautifully as was the case in our patient.[3] Once diagnosed appendectomy followed by adequate drainage of the abscess is the best treatment for the retroperitoneal abscess resulting from the ruptured retrocecal appendicitis.[2,8,7]

We conclude that the formation of complicated retroperitoneal abscesses involving the perinephric space is a rare but serious complication of perforated acute appendicitis. Such an intra-abdominal pathological abnormality cannot be excluded in a patient presenting without abdominal symptoms. The mortality rate can only be reduced by a high index of suspicion, early and accurate diagnosis ideally with MDCT, and appropriate surgical treatment.
